# Designed Mutations Alter the Binding Pathways of an Intrinsically Disordered Protein

**DOI:** 10.1038/s41598-019-42717-6

**Published:** 2019-04-16

**Authors:** Di Wu, Huan-Xiang Zhou

**Affiliations:** 1 Department of Physics and Institute of Molecular Biophysics, Florida State University, Tallahassee, FL 32306 USA; 2 Department of Chemistry and Department of Physics, University of Illinois at Chicago, Chicago, IL 60607 USA

## Abstract

Many cellular functions, including signaling and regulation, are carried out by intrinsically disordered proteins (IDPs) binding to their targets. Experimental and computational studies have now significantly advanced our understanding of these binding processes. In particular, IDPs that become structured upon binding typically follow a dock-and-coalesce mechanism, whereby the docking of one IDP segment initiates the process, followed by on-target coalescence of remaining IDP segments. Multiple dock-and-coalesce pathways may exist, but one may dominate, by relying on electrostatic attraction and molecular flexibility for fast docking and fast coalescing, respectively. Here we critically test this mechanistic understanding by designing mutations that alter the dominant pathway. This achievement marks an important step toward precisely manipulating IDP functions.

## Introduction

Intrinsically disordered proteins (IDPs) and proteins containing intrinsically disordered regions (IDRs) account for nearly one half of all proteins^1^. The disordered nature allows these proteins to facilely adapt to different interaction partners, making them well suited for signaling and regulation in a variety of cellular processes^2–5^. While some IDPs remain disordered upon binding partners^6^, others undergo a disorder-to-order transition upon binding structured targets. Even in the latter case, IDPs differ from typical structured proteins in characteristic ways in the structures formed, in the interactions stabilizing the complexes, and in the kinetics of the binding processes. Experimental and computational studies together have significantly advanced the understanding on these characteristics, and now critical testing becomes timely. Protein design has been an important approach for such testing for structured proteins, but its use on IDPs has been very limited. This includes the modulation of IDP compactness by changing charge patterning along the sequence^7–9^, the gain of structure by introducing mutations into an IDP^10^, and the design of antibodies that target specific epitopes within IDPs^11^. The present study aimed to design mutations that would alter binding pathways of IDPs in a predicted manner.

When IDPs form structures upon binding to their targets, the structures often are highly extended and slender, with stability largely provided by intermolecular rather than intramolecular interactions^2,12–15^. The resulting interfaces thus lack a well-defined hydrophobic core region as typically found in complexes of structured proteins. Correspondingly charge-charge and polar interactions play a greater role in stabilizing the complexes between IDPs and their targets^14^, although there are also complexes of structured proteins where electrostatic interactions are a significant stabilizing factor^16,17^. The extended structure of a bound IDP and the high proportion of charged and polar groups in interfacial residues, along with fast adaptive reconfiguration of the IDP during its binding process, also impact binding kinetics, both in the pathways leading to the native complex and in the magnitude of the association rate constant (*k*_a_). In particular, it is likely that the folding upon binding process involves intermediates in which only a part of the IDP is engaged with the target, as suggested in a number of computational studies^18–28^. Experimental studies have produced evidence for the existence of such intermediates^29–36^.

**Figure 1:**
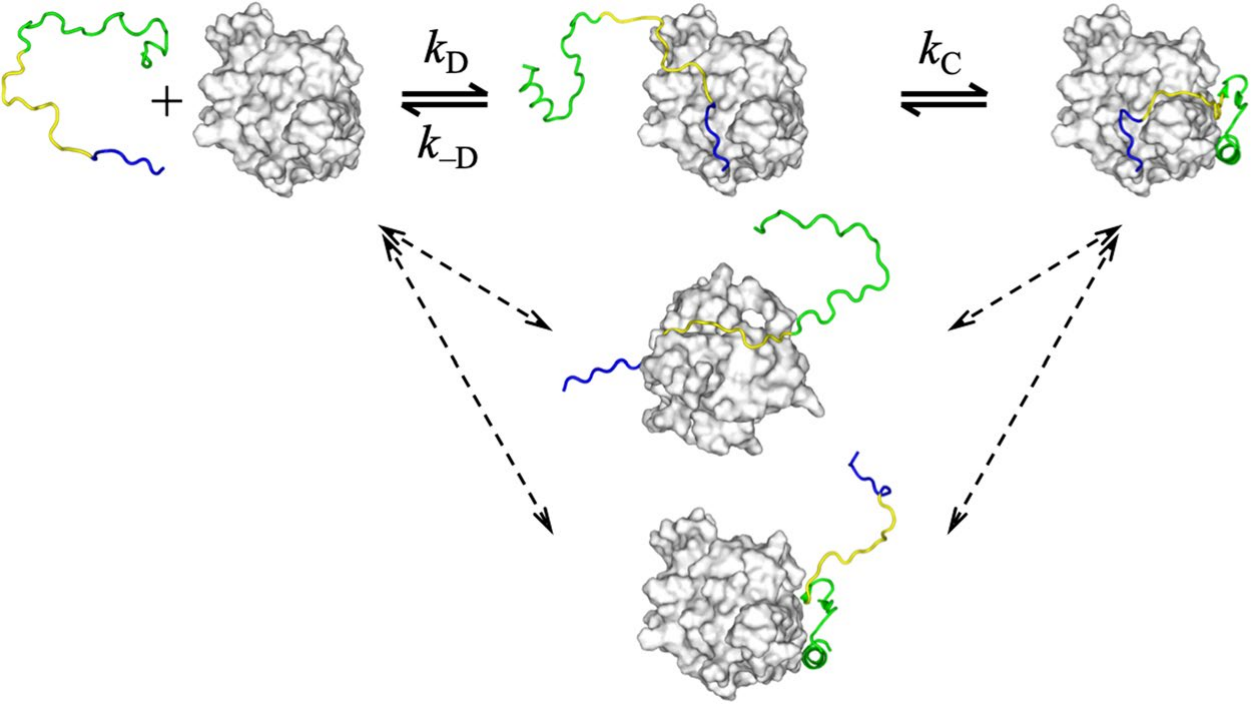
Multiple pathways for the binding of an intrinsically disordered protein (IDP) to a structured target. Each pathway is initiated by the docking of an IDP segment, followed by the coalescence of the remaining IDP segments around their respective subsites on the target surface.

If the partially engaged intermediates are on-pathway, then the coupled folding and binding occur via a sequential mechanism. We have proposed a particular flavor of sequential mechanism called dock-and-coalesce, whereby a segment of an IDP first docks to its cognate subsite on the target, leading to an intermediate referred to as the partially docked complex; tethered to the docked segment, other segments of the IDP then coalesce to their respective subsites (Fig. 1)^24^. This mechanism corresponds to the following kinetic scheme:

1 $${\rm{D}}+{\rm{T}}\begin{array}{c}{k}_{{\rm{D}}}\\ \,\rightleftharpoons \,\\ {k}_{-{\rm{D}}}\end{array}{\rm{D}}\cdot {\rm{T}}\,\mathop{\rightleftharpoons }\limits^{{k}_{{\rm{C}}}}\,{\rm{DT}}$$

where D, T, D·T, and DT represent the IDP, target, partially docked complex, and native complex, respectively, *k*_D_ is the rate constant to form the partially docked complex, and *k*_–D_ and *k*_C_ are the rate constants for this intermediate to either undock or coalesce into the native complex. If the partially docked complex is only very transiently formed, then the overall association rate constant is

2 $${k}_{{\rm{a}}}=\frac{{k}_{{\rm{D}}}{k}_{{\rm{C}}}}{{k}_{-{\rm{D}}}+{k}_{{\rm{C}}}}=\frac{{k}_{{\rm{D}}}}{1+{k}_{-{\rm{D}}}/{k}_{{\rm{C}}}}$$

Note that the docking rate constant *k*_D_ sets an upper bound for *k*_a_; the ratio of *k*_a_ and *k*_D_ is *k*_C_/(*k*_–D_ + *k*_C_), which is the committer for coalescence. Slow coalescence or fast undocking leads to a reduced committer and hence a reduced *k*_a_.

**Figure 2:**
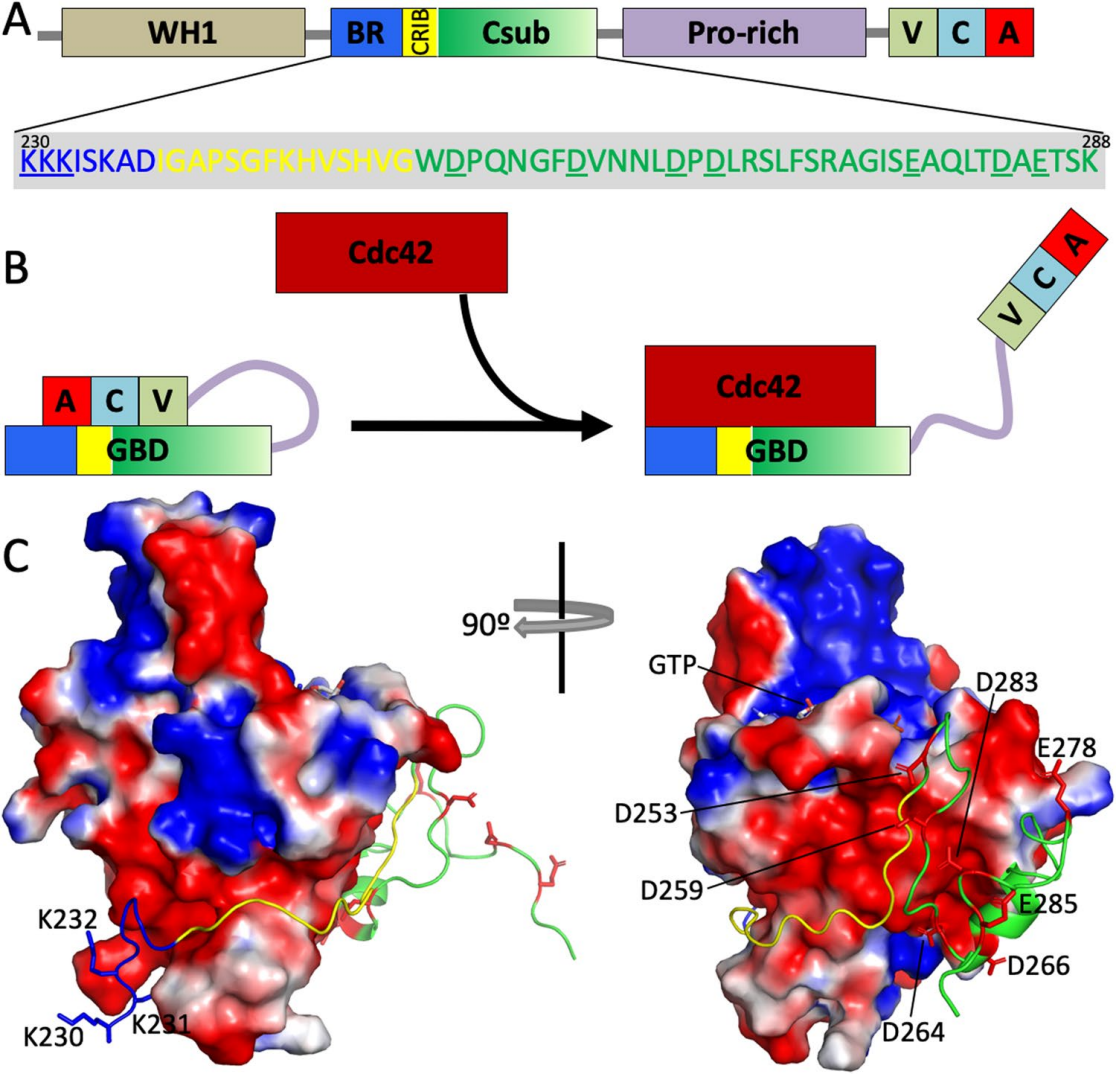
Domain organization of the Wiskott-Aldrich Syndrome protein (WASP) and its interaction with Cdc42. (**A**) Domain organization of WASP. The GTPase binding domain (GBD) can be further divided into a basic region (BR), a CRIB motif, and a C-terminal subdomain (Csub). Three cationic residues in BR and seven anionic residues in Csub are underlined in the sequence. (**B**) Autoinhibition of WASP, via intramolecular binding between GBD and VCA domains, and activation by binding with the Cdc42 GTPase, thereby releasing the VCA domains for downstream signaling. (**C**) Structure and electrostatic interactions of the GBD-Cdc42 complex (Protein Data Bank entry 1CEE). Cdc42 is shown by its electrostatic surface (blue and red colors represent positive and negative electrostatic potentials, respectively). GBD is shown as cartoon, with BR, CRIB, and Csub in blue, yellow, and green, respectively. Cationic side chains in BR and anionic side chains in Csub are shown as stick.

The binding of many IDPs appears to follow some form of dock-and-coalesce^13^. One example is the binding of the GTPase-binding domain (GBD), an IDR, of the Wiskott-Aldrich Syndrome protein (WASP) to the Cdc42 GTPase (Fig. 2). The bound conformation of GBD^37^, in keeping with other IDPs, consists of a highly extended N-terminal basic region (BR) and middle CRIB motif, as well as a relatively compact C-terminal subdomain (Csub). Regular secondary structures, including an α-helix consisting of residues Pro265 to Ser277, occur only in Csub. Kinetic and computational studies have suggested that, in the protein concentration range where the binding rate is proportional to concentration, the binding process is initiated and largely rate-limited by the docking of GBD BR; the subsequent on-target coalescence of the remaining GBD segments is relatively fast^26,31^. The measured *k*_a_ reached 22 μM^−1^ s^−1^ at low salt and decreased significantly by adding salt and by mutating cationic residues in GBD BR or anionic residues in the corresponding subsite on Cdc42^31^. The computed rate constant for docking BR, *k*_D_, was 33 μM^−1^ s^−1^, only slightly higher than the observed overall rate constant *k*_a_; moreover, the computed effects of salt and charge mutations mirrored those observed on *k*_a_^26^. One can thus conclude that not only BR docking is largely rate-limiting for GBD binding, but also this docking rate is significantly accelerated by electrostatic attraction.

To further dissect the relative contributions of the docking and coalescing steps, we differentially perturbed these two steps by changing temperature and adding glucose or urea^38^. These data suggest that the committer for coalesce, involving CRIB and Csub of GBD, is approximately 2/3 and hence *k*_a_ is only 1.5-fold smaller than the BR docking rate constant. Thus, despite large-scale GBD conformational changes involved, including secondary structure formation in Csub, the coalescing step, facilitated by intrinsic flexibility or secondary structure propensity and by interactions with the target, can be fast and avoid becoming rate-limiting to the overall binding process. Similar findings have been obtained on the binding of other IDPs^13,22,25,33,39–43^. For IDPs in isolation, rapid reconfiguration (on timescales of 100 ns) has been observed by nanosecond fluorescence correlation spectroscopy combined with single-molecule fluorescence resonance energy transfer^44^.

Potentially any part of the IDP can act as the docking segment and initiate a dock-and-coalesce pathway, and therefore multiple pathways may exist^13,15,26,27,43,45^ (Fig. 1). Still, one pathway may dominate, when the association rate constant through that pathway is much higher than through all other pathways. The most likely dominant pathway is the one where the docking step is rate-enhanced by electrostatic attraction and the coalescing step is sufficiently fast as not to be rate-limiting. For WASP GBD binding to Cdc42, as noted above, the BR-initiated pathway has been established as the dominant pathway^26,31,38^. According to our computation, the docking rate constants of the pathways initiated by BR, CRIB, and Csub are 33, 0.17, and 0.019 μM^−1^ s^−1^, respectively, and the differences stem from the degree of electrostatic complementarity with the target^26^. Cdc42 has a mostly negative electrostatic surface, whereas BR and Csub are enriched in cationic and anionic residues, respectively, and CRIB is largely free of charged residues (Fig. 2A,C). Hence electrostatic interactions accelerate the BR-initiated pathway, retard the Csub-initiated pathway, and have little effect on the CRIB-initiated pathway. The observation that electrostatic interactions can dictate the dominant binding pathway presents a unique opportunity for using charge mutations to alter the relative contributions of different binding pathways to the overall *k*_a_.

Here we exploit this opportunity, by introducing GBD mutations to slow down the BR-initiated pathway along with Cdc42 mutations to accelerate the Csub-initiated pathway. The GBD mutations were to neutralize charges in BR, with Lys230Lys231Lys232 substituted by Ala residues (construct known as GBD_3A_), whereas the Cdc42 mutations were to remove negative charges and add positive charges around the subsite for Csub, with Glu31, Asn39, Glu62, Asp63, Asp65, and Gln74 all substituted by Lys residues (construct known as Cdc42_6K_). Stopped-flow measurements showed that the rate constant of GBD_3A_ was lower by 6-fold than wild-type GBD (GBD_WT_) for binding to wild-type Cdc42 (Cdc42_WT_). However, upon introducing the 6K mutations on Cdc42 as well, the loss in *k*_a_ was more than recovered, with *k*_a_ for the mutant proteins, GBD_3A_ and Cdc42_6K_, 2-fold higher than the wild-type proteins. The design goal of promoting the Csub-initiated pathway into the dominant one is thus achieved by the introduced mutations. Further evidence for the new dominant pathway is provided by effects of salt and urea. Lastly, in the binding of GBD_WT_ with Cdc42_6K_, both the BR-initiated and the Csub-initiated pathways appear to be prominent, with the measured rate constant close to the sum of the contributions from the two pathways.

## Results

Our stopped-flow measurement yielded a *k*_a_ of (5.53 ± 0.27) μM^−1^ s^−1^ for the binding of wild-type GBD and Cdc42 in 50 mM sodium phosphate buffer at pH 7.0 and 25 °C (Figs S1A and S2). This *k*_a_ value is somewhat lower than those obtained previously^31,38^, due to the shorter GBD construct used here (residues 228–298 here compared to residues 154–322 in earlier studies). The dissociation constant (*K*_d_) determined here is 0.114 ± 0.009 μM. From *k*_a_ and *K*_d_, the deduced dissociation rate constant (*k*_d_ = *k*_a_*K*_d_) is 0.63 s^−1^, which agrees closely with the value, 0.696 ± 0.011 s^−1^, measured directly by a competition assay (Fig. S1B). Below we show how these kinetic and thermodynamic constants are affected by mutations designed to alter binding pathways.

### Slowdown of the original dominant pathway by removing BR cationic residues

**Figure 3:**
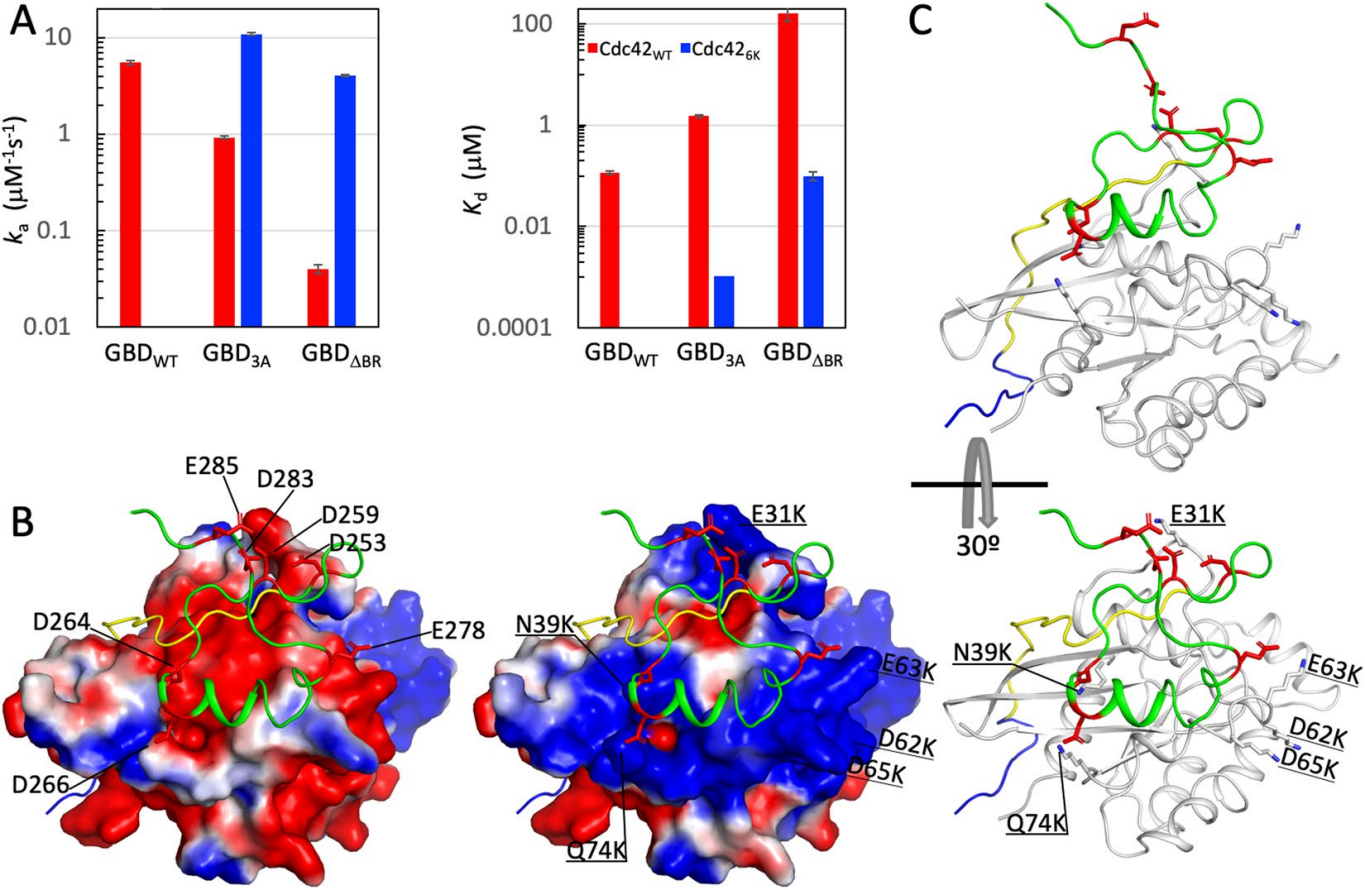
GBD and Cdc42 mutations and their effects on binding kinetic and thermodynamic properties. (**A**) Association rate constants (*k*_a_; left panel) and dissociation constants (*K*_d_; right panel) for three GBD constructs, GBD_WT_, GBD_3A_, and GBD_ΔBR_, interacting with either Cdc42_WT_ (red bars) or Cdc42_6K_ (blue bars). (**B**) Comparison of electrostatic surfaces of Cdc42_WT_ (left panel) and Cdc42_6K_ (right panel) around the subsite for GBD Csub. Seven anionic residues in Csub are shown as stick and labeled in the left panel. The six Lys mutations in Cdc42_6K_ are indicated by underlined labels in the right panel. (**C**) Two views of the complex between GBD and Cdc42_6K_, with both proteins as cartoon, to indicate that the 6K mutation sites are at the periphery of the subsite for Csub.

The first set of mutations, substituting Lys230Lys231Lys232 in GBD BR by Ala residues, was designed to slow down the BR-initiated pathway, which has previously been established as being dominant in the binding of the wild-type proteins^26,31,38^. Consistent with the dominant role of BR, the binding of GBD_3A_ with Cdc42_WT_ was slowed down by 6-fold, to (0.92 ± 0.03) μM^−1^ s^−1^ (Figs 3A and S2). Our previous computational study^26^ found that the 3A mutations reduced the BR-initiated docking rate constant by 117-fold, from 33 μM^−1^ s^−1^ to 0.28 μM^−1^ s^−1^. The latter is still higher than but has the same order of magnitude as the CRIB-initiated docking rate constant (at 0.17 μM^−1^ s^−1^). So both the BR-initiated and the CRIB-initiated pathways can be assumed to be significant in the binding of GBD_3A_ with Cdc42_WT_.

To further suppress the BR-initiated pathway, we changed Lys230Lys231Lys232 in GBD BR to Glu residues (GBD_3E_), thereby turning electrostatic attraction with Cdc42 into repulsion. Correspondingly, the calculated docking rate constant for the BR-initiated pathway of this mutant is very small, at 0.002 μM^−1^ s^−1^ ^26^. Our measured *k*_a_ for GBD_3E_ was (0.097 ± 0.013) μM^−1^ s^−1^ (Fig. S2), which is approximately 60-fold lower than the counterpart for GBD_WT_ but 50-fold higher than the docking rate constant for the BR-initiated pathway of GBD_3E_. The latter result indicates that an alternative pathway has become dominant. The most likely candidate is the CRIB-initiated pathway, which has a docking rate constant just slightly higher than the observed *k*_a_ for GBD_3E_. This small difference can be attributed to a less than unity committer for coalescence in the CRIB-initiated pathway, or perhaps the anionic residues in GBD_3E_ BR have a hindrance effect on the docking step of the CRIB-initiated pathway.

We also made a GBD construct (residues 238–298; GBD_ΔBR_) where the BR segment was deleted, to completely eliminate the BR-initiated pathway. Its association rate constant was (0.040 ± 0.004) μM^−1^ s^−1^ (Figs 3A and S2). This value is approximately 140-fold lower than the counterpart for GBD_WT_, but higher than the docking rate constant of the Csub-initiated pathway. So here again the CRIB-initiated pathway is likely to be dominant.

In comparison to the 6-, 60- and 140-fold reductions in *k*_a_ for the GBD_3A_, GBD_3E_, and GBD_ΔBR_ mutants (relative to GBD_WT_), the changes in *k*_d_ were less drastic, with increases of 2-, 7-, and 10-fold, respectively. With the combined effects on *k*_a_ and *k*_d_, the 3A mutation and BR deletion, respectively, led to 13- and 1421-fold weakening in binding affinity (Fig. 3A).

### Promotion of a new dominant pathway by introducing cationic residues around the subsite for Csub

The foregoing results show that it is relatively easy to introduce electrostatic repulsion (as in GBD_3E_) to slow down a dominant pathway sufficiently so that a lesser pathway becomes dominant. The cost is that the overall association rate constant is significantly reduced. We aimed to design mutations that not only altered the dominant pathway but also maintained or even exceeded the association rate constant of the wild-type proteins.

Our initial attempt was to make additional mutations on the Csub segment of the GBD_3A_ background. While Cdc42 has a mostly negative electrostatic surface, there is a small positive patch around the subsite for Csub, due to residue Arg66 of Cdc42 (Fig. S3A; Cdc42 residues underlined in order to distinguish from GBD residues). In the native complex, next to Arg66 are two GBD residues, Arg273 and Gly275, toward the C-terminus of the Csub α-helix. We mutated these two residues into Glu and Asp (GBD_3A/ED_), respectively, hoping that they would form favorable electrostatic interactions with Cdc42 Arg66. However, instead of a rate enhancement, the ED mutations reduced *k*_a_ by 2.5-fold and raised *K*_d_ by 3.7-fold (Figs S2 and S3B). Possibly any electrostatic attraction between the introduced anionic residues with Cdc42 Arg66 was outweighed by repulsion with the overall negative electrostatic surface of Cdc42 around the subsite for Csub. Another possibility is that the ED mutations perturbed the secondary structure propensities of GBD, as residual secondary structures have been shown to be a rate determinant of IDP binding^33,38,40,42^. Indeed, while the 3A mutations mostly preserved the circular dichroism (CD) spectrum of GBD_WT_, the additional ED mutations led to an approximately 50% decrease in the magnitude of the CD spectrum at 222 nm, indicating a significant reduction in residual α-helix content (Fig. S3C). In addition to GBD_3A_, GBD_ΔBR_ also had a CD spectrum closing matching that of GBD_WT_. Together the CD data confirmed that residual secondary structures were confined to the Csub sequence.

To avoid any complication from perturbations of GBD secondary structure propensities and also to take advantage of the high content of anionic residues in Csub (Fig. 2C), we decided to make mutations on Cdc42 that would turn the negative electrostatic surface around the subsite for Csub into a positive one (Fig. 3B). We chose six residues on the rim of the subsite and mutated them to cationic Lys, including: Glu31, which is close to Csub Asp253 and Asp259; a cluster of three anionic residues, Glu62, Asp63, and Asp65, which are close to Glu278 following the Csub α-helix; and Asn39 and Gln74, which are close to Asp264 and Asp266 at the N-terminus the Csub α-helix. These mutations were located away from the direct interface of the native complex, and thus were expected to mostly introduce long-range electrostatic interactions and have less influence on other short-range interactions that stabilize the native complex (Fig. 3C). The surface mutations also did not affect the secondary structures of Cdc42, as judged by CD spectra (Fig. S4).

The 6K mutations on Cdc42 boosted the association rate constant of GBD_3A_ by 12-fold, to (10.9 ± 0.3) μM^−1^ s^−1^, which is even higher than the *k*_a_ of the wild-type proteins by 2-fold (Figs 3A and S2). This large increase in *k*_a_, achieved by new electrostatic attraction of Csub, suggests that the Csub-initiated pathway now becomes dominant. As support for the new dominant pathway, we found that the 6K mutations on Cdc42 were even able to increase the *k*_a_ of GBD_ΔBR_ to a level comparable to those for GBD_3A_-Cdc42_6K_ and GBD_WT_-Cdc42_WT_ binding (Figs 3A and S2). The rate constant, (4.03 ± 0.08) μM^−1^ s^−1^, for GBD_ΔBR_-Cdc42_6K_ binding is 100-fold higher than that for GBD_ΔBR_-Cdc42_WT_ binding. Since GBD_ΔBR_ lacks the BR segment but its Csub benefits from the same electrostatic attraction by Cdc42_6K_ as the Csub of GBD_3A_, the most logical explanation for the comparable rate constants is that the Csub-initiated pathway is dominant for both GBD_ΔBR_ and GBD_3A_ in binding to Cdc42_6K_.

The 6K mutations on Cdc42 also decreased the dissociation rate constants of GBD_ΔBR_ and GBD_3A_, by 16- and 121-fold, respectively. Combined with the effects on *k*_a_, the 6K mutations led to an approximately 1500-fold increase in binding affinity for both GBD_ΔBR_ and GBD_3A_ (Fig. 3A). As a result, the GBD_ΔBR_-Cdc42_6K_ affinity is even slightly higher than that of the wild-type proteins, and the GBD_3A_-Cdc42_6K_ affinity is higher by approximately 100-fold than the wild-type counterpart.

### Effects of salt and urea corroborate the new dominant pathway

**Figure 4:**
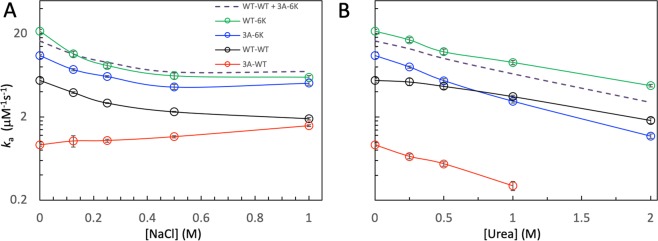
Effects of NaCl and urea on the association rate constants of four GBD-Cdc42 pairs. (**A**) Dependence on NaCl concentration. (**B**) Dependence on urea concentration. The dashed curves are rate constants predicted for the GBD_WT_-Cdc42_6K_ pair, as the sum of the rate constants for the GBD_WT_-Cdc42_WT_ and GBD_3A_-Cdc42_6K_ pairs.

Perturbation of solvent conditions, e.g., through the addition of cosolvents, has been very useful for gaining insight into binding mechanisms^38,39,41^. Here we used NaCl and urea to probe electrostatic interactions and secondary structure formation in the binding of the different GBD and Cdc42 constructs. In line with previous studies^31,38^ and with significant electrostatic attraction between GBD BR and Cdc42, the *k*_a_ of the wild-type proteins decreased significantly by the addition of NaCl, which screens electrostatic interactions (Fig. 4A). Interestingly, with the neutralization of the cationic residues in GBD BR by the 3A mutations, *k*_a_ no longer decreased with increasing salt but actually showed a slight increase. The latter trend is likely a reflection of the electrostatic repulsion between GBD Csub and its subsite on Cdc42. Through charge reversal of the subsite for Csub by the 6K mutations, *k*_a_ again decreased significantly with increasing NaCl concentration, suggesting that now the docking step of the Csub-initiated pathway is largely rate-limiting for the overall binding process.

As shown previously^38^, addition of urea slowed down the binding of wild-type GBD and Cdc42, by hindering Csub secondary structure formation in the coalescing step of the BR-initiated pathway. Our data here show that urea had a stronger effect on the *k*_a_ of GBD_3A_-Cdc42_WT_ binding than on the wild-type *k*_a_ (Figs 4B, S5A,B). 1 M urea decreased the association rate constants by 1.6-fold for GBD_WT_ but 3.1-fold for GBD_3A_. The 3A mutations may reduce the committer for coalescence in the BR-initiated pathway, leading to a greater rate-determining role for the coalescing step of GBD_3A_ and thereby explaining the stronger urea effect on its *k*_a_. Urea had an even stronger effect on the *k*_a_ of GBD_3A_-Cdc42_6K_ binding, with decreases by 3.5- and 9.2-fold, respectively, at 1 and 2 M urea (Figs 4B, S5C and S6A). This very strong urea effect is expected, since hindrance of secondary structure formation now slows down Csub docking, which as we contend is rate-limiting for the GBD_3A_-Cdc42_6K_ binding.

### Both BR- and Csub-initiated pathways are significant for GBD_WT_-Cdc42_6K_ binding

We wondered what pathways the binding of GBD_WT_ and Cdc42_6K_ would follow. This pair of proteins should have strong electrostatic attraction when either GBD BR or Csub docks to its subsite. Therefore both BR- and Csub-initiated pathways might be significant in this case. Then the overall association rate constant should be the sum of contributions from the two parallel pathways. The GBD_WT_-Cdc42_WT_ binding is dominated by the BR-initiated pathway and may serve as a surrogate for the BR-initiated pathway for GBD_WT_-Cdc42_6K_ binding. Likewise the GBD_3A_-Cdc42_6K_ binding may serve as a surrogate for the Csub-initiated pathway for GBD_WT_-Cdc42_6K_ binding. We can thus predict the *k*_a_ for GBD_WT_-Cdc42_6K_ binding as the sum of the *k*_a_ values for GBD_WT_-Cdc42_WT_ and GBD_3A_-Cdc42_6K_ binding, which would be 16.4 μM^−1^ s^−1^ in buffer. This prediction only slightly underestimates the observed value, (21.4 ± 1.2) μM^−1^ s^−1^, for GBD_WT_-Cdc42_6K_ binding (Fig. S2). Moreover, this additive prediction, shown as dashed curves in Fig. 4 (see also Fig. S5D), agrees well with the measured values for GBD_WT_-Cdc42_6K_ binding over the entire ranges of NaCl and urea concentrations.

### Mutant proteins preserve the wild-type binding pose

In contrast to the disparate dependences of *k*_a_ on urea concentration among the GBD_WT_-Cdc42_WT_, GBD_3A_-Cdc42_WT_, and GBD_3A_-Cdc42_6K_ pairs noted and explained above (Fig. 4B), the dependences of the equilibrium dissociation constant, *K*_d_, are similar (Fig. S6B). ln*K*_d_ had a nearly linear dependence on [Urea], with slopes of 1.6, 1.6, and 2.2 M^−1^ for the three protein pairs. In protein folding equilibrium, the corresponding slope has been interpreted as a measure of the surface area buried upon folding^46^. Based on an analogous interpretation, the urea-dependent *K*_d_ data hint that the bound complexes of the wild-type and two mutant pairs have nearly the same buried surface area at their interfaces.

**Figure 5:**
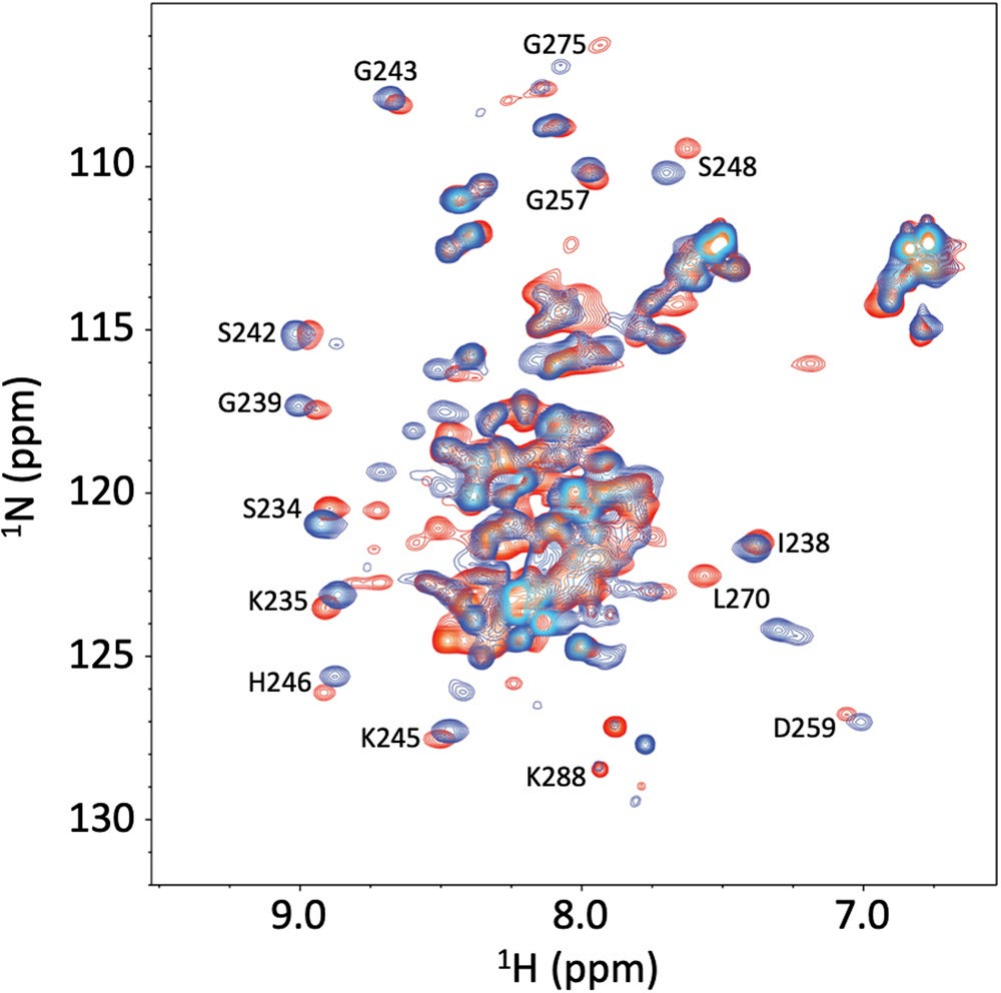
^1^H-^15^N HSQC spectra of GBD_WT_ (red) bound to Cdc42_WT_ and GBD_3A_ bound to Cdc42_6K_ (blue).

To obtain further evidence that the GBD_3A_-Cdc42_6K_ complex preserved the wild-type binding pose, we acquired ^1^H-^15^N HSQC spectra of GBD_3A_ bound to Cdc42_6K_ and GBD_WT_ bound to Cdc42_WT_ (Fig. 5). Most of the more dispersed GBD_3A_ resonances, which are either structured or otherwise involved in interactions with Cdc42_6K_, can be unequivocally identified based on their proximity to assigned resonances of GBD_WT_ bound to Cdc42_WT_^37^. These include Ser234 and Lys235 in BR, Ile238, Gly239, Ser242, Gly243, Lys245, His246, and Ser248 in CRIB, and Gly257, Asp259, and Lys288 in Csub (Fig. S7). The proximity of the corresponding resonances of bound GBD_3A_ and bound GBD_WT_ indicates that the two complexes have the same binding pose.

## Discussion

In previous work we have proposed dock-and-coalesce as a general mechanism for the binding of IDPs that form extended conformations on structured targets, and for the WASP GBD-Cdc42 pair have characterized the precise form of dock-and-coalesce. These and other studies have led to the conclusion that multiple dock-and-coalesce pathways exist and the dominant pathway is dictated by electrostatic attraction mediated by the specific docking segment. Here we have tested this mechanistic understanding by introducing charge mutations to alter binding pathways in a predicted manner. The mutations were designed to slow down one pathway and to accelerate another pathway, so that the overall association rate constant is maintained but the dominant pathway is switched. This design goal has been reached, as documented by several lines of evidence. First, the measured rate constants changed precisely according to design, with the 3A mutations on GBD BR reducing the rate constant by 6-fold, but the 6K mutations on Cdc42 around the subsite for GBD Csub bringing the rate constant back up by 12-fold. Second, the effects of salt and urea were just what were expected if the dominant pathway switched from the BR-initiated one to the Csub-initiated one. Third, as predicted, both the BR- and Csub-initiated pathways were significant for the binding of GBD_WT_ with Cdc42_6K_. Lastly, NMR data indicated that, while the 3A and 6K mutations altered the binding pathways, they preserved the structure of the final complex.

The design strategy illustrated here on the WASP GBD-Cdc42 pair can be directly applied to many other IDPs that form highly extended conformations on their target surfaces. More broadly, it seems inevitable that the coupled folding and binding of IDPs involve multiple pathways^43^. By tuning down rate-determining factors in some pathways and tuning up these factors in other pathways, one can methodically manipulate the binding mechanisms. For the WASP GBD-Cdc42 pair, we have identified electrostatic attraction as the predominant rate-determining factor. Other possible rate-determining factors include secondary structure propensities of IDPs and short-range interactions with their targets.

Our design strategy has been framed on the basis that there is a fixed set of parallel binding pathways, and mutations only affect the relative weights of these pathways. It is possible that some mutations open entirely new pathways. While we could not rule out this possibility for the mutations introduced in the present study, there was no evidence for it either. Nevertheless this caveat should be kept in mind when extending this design strategy to other applications.

Design has been a very valuable approach in testing our understanding of structure, stability, kinetics, and function for structured proteins. For IDPs, design studies have been limited. These include the modulation of IDP compactness by changing charge patterning along the sequence^7–9^, the gain of structure by introducing mutations into an IDP^10^, and the design of antibodies that target specific epitopes within IDPs^11^. Much like our strategy of using charge mutations for altering binding mechanisms, scrambling of charges along the sequence has predictable effects on IDP size, with segregation of opposite charges leading to compaction. The effects of other residues such as Pro and aromatic ones are context-dependent and hence less generalizable as the basis of design strategies.

IDPs usually have multiple interaction partners. The existence of multiple pathways of an IDP when binding with a single partner both adds to the complexity of the signaling networks but also presents new opportunities for manipulating the outcome of the signaling networks. For example, different pathways in binding a partner may produce intermediates that preferentially favor interactions with different downstream effectors. By accelerating one pathway, signaling via a specific effector may be promoted. As another example, an IDP may have several phosphorylation sites, and the order of phosphorylation may be functionally important. How such order is achieved may teach us strategies for future designs of IDP binding mechanisms.

## Methods

### Plasmids

Human Cdc42 (Genbank accession number nm_001791; residues 1−179), human WASP (Genbank nm_000377; residues 228–298, construct referred to as GBD) were cloned into pDest-527 (with a His6 tag) and pGEX 2 T (with GST fusion) expression vectors, respectively. Mutations in Cdc42 and GBD were obtained by PCR mutagenesis. The truncated GBD (residues 238–298, GBD_ΔBR_) was obtained by PCR from the plasmid of GBD.

### Protein preparation

The protocols used largely followed those described previously^38^. All proteins were expressed in *Escherichia coli* Rosetta cells. Cdc42 constructs were first purified through a HisTrap column. The His6 tag was then removed by in-house expressed His6-tagged TEV protease; four extra residues (Gly-Ser-Phe-Thr) from the expression vector were left at the N-terminus of Cdc42. The solution passed through the HisTrap column one more time to remove any His6-tagged components. GBD constructs were first purified through a GSTrap column. The GST tag was removed by thrombin (catalog number 154163, MP Biomedicals, Santa Ana, CA); two extra residues (Gly-Ser) from the thrombin recognition site in the fusion construct were left at the N-terminus of GBD. Subsequently the solution was incubated at 70 °C for 20 min to induce aggregation of structured proteins; the disordered GBD constructs were collected in the supernatant after centrifugation at 20,000 rpm for 30 min. All Cdc42 and GBD constructs went through a final purification step through a Superdex 75 gel filtration column. All columns were from GE Healthcare Life Sciences (Pittsburgh, PA).

All Cdc42 constructs were loaded with GppNHp (guanosine 5′-β,γ-imidotriphosphate; Sigma-Aldrich St. Louis, MO) or mantGppNHp (2′,3′-O-N-methylanthraniloyl-GppNHp; Jena Bioscience, Germany), prepared as described^31,38^. The Cdc42 species loaded with these GTP analogs, one nonfluorescent and the other fluorescent, are denoted as GppNHp·Cdc42 and mantGppNHp·Cdc42, respectively.

### Stopped-flow fluorescence spectroscopy

Binding rate constants and affinities were measured by monitoring the fluorescence intensity of mantGppNHp·Cdc42 on a stopped-flow instrument (SX20, Applied Photophysics, UK)^38^. The excitation wavelength was 366 nm and emission was collected with a cutoff filter at 395 nm. All measurements were made in 50 mM sodium phosphate buffer at pH 7.0 with 5 mM MgCl_2_ and 1 mM TECP at 25 °C. The mantGppNHp·Cdc42 concentration, [Cdc42], was always maintained at 0.1 μM in one syringe.

For *k*_a_ measurements, the GBD concentration, [GBD], was in excess in a second syringe. Following mixing of the two proteins, the time-dependent fluorescence intensity was fitted to a single exponential, leading to an observed rate *k*_obs_ (Fig. S1A,C). For each [GBD], three to six replicate measurements were made for *k*_obs_, and for a given pair of Cdc42 and GBD constructs, *k*_obs_ was determined at five or more [GBD] values. The relation between *k*_obs_ values and [GBD] values was fitted to

3 $${k}_{{\rm{obs}}}={k}_{{\rm{a}}}[{\rm{GBD}}]+{k}_{{\rm{d}}}$$

(Figs. S2 and S5). Fitting errors for *k*_a_ were reported as errors. Though this fit in theory also yielded the dissociation rate constant *k*_d_, the values were unreliable because they were small and could be overwhelmed by fitting errors. Instead, one or both of two other methods were used for measuring *k*_d_.

The first alternative was to determine the dissociation constant *K*_d_ and then deduce *k*_d_ as the product of *K*_d_ and *k*_a_. For *K*_d_ determination, we used the difference, Δ*I*, between the fluorescence intensity before mixing and that after reaching equilibrium. The Δ*I* values at seven [GBD] values were fitted to a binding model:

4 $${\rm{\Delta }}I={\rm{\Delta }}{I}_{{\rm{t}}}\frac{[{\rm{Cdc}}42]+[{\rm{GBD}}]+{K}_{{\rm{d}}}-\sqrt{{([{\rm{Cdc}}42]+[{\rm{GBD}}]+{K}_{{\rm{d}}})}^{2}-4[{\rm{Cdc}}42][{\rm{GBD}}]}}{2[{\rm{Cdc}}42]}$$

where ${\rm{\Delta }}{I}_{{\rm{t}}}$ is the difference in fluoresce intensity between unbound and bound mantGppNHp·Cdc42.

*k*_d_ was also directly measured by displacing mantGppNHp·Cdc42 complexed with GBD by a high excess of GppNHp·Cdc42. In these measurements, 0.1 μM mantGppNHp·Cdc42 and 1 μM GBD in one syringe were mixed with 20 μM GppNHp·Cdc42 in a second syringe. The time-dependent fluorescence intensity was fitted to a single exponential to yield *k*_d_ (Fig. S1B).

### NMR spectroscopy

^1^H-^15^N HSQC spectra of ^15^N labeled GBD_WT_ and GBD_3A_ at 1.2 mM mixed with Cdc42_WT_ and Cdc42_6K_, respectively, at 1.44 mM were obtained on a Bruker 800 MHz spectrometer at 25 °C. Resonance assignment of bound GBD_WT_ was from Rosen and co-workers^37^.

### Circular dichroism spectroscopy

Proteins at 10 μM in a 1 mm path length cuvette were scanned on a CD spectrometer (Model 420, Aviv, Lakewood, NJ). The average of five scans was reported.

## Supplementary information

Supplementary Information
