# Supplementary material for: Designed Mutations Alter the Binding Pathways of an Intrinsically Disordered Protein
Source: Sci Rep. 2019 Apr 16;9:6172. doi: 10.1038/s41598-019-42717-6 (PMC6467919; doi:10.1038/s41598-019-42717-6)
Supplement: Supplementary file 1 — Supplementary Information [file 41598_2019_42717_MOESM1_ESM.pdf]

Designed Mutations Alter the Binding Pathways of an Intrinsically Disordered Protein

Di Wu[1] and Huan-Xiang Zhou[2,*]

[1]Department of Physics and Institute of Molecular Biophysics, Florida State University, Tallahassee, FL 32306, USA

[2]Department of Chemistry and Department of Physics, University of Illinois at Chicago, Chicago, IL 60607, USA

Supplementary Information

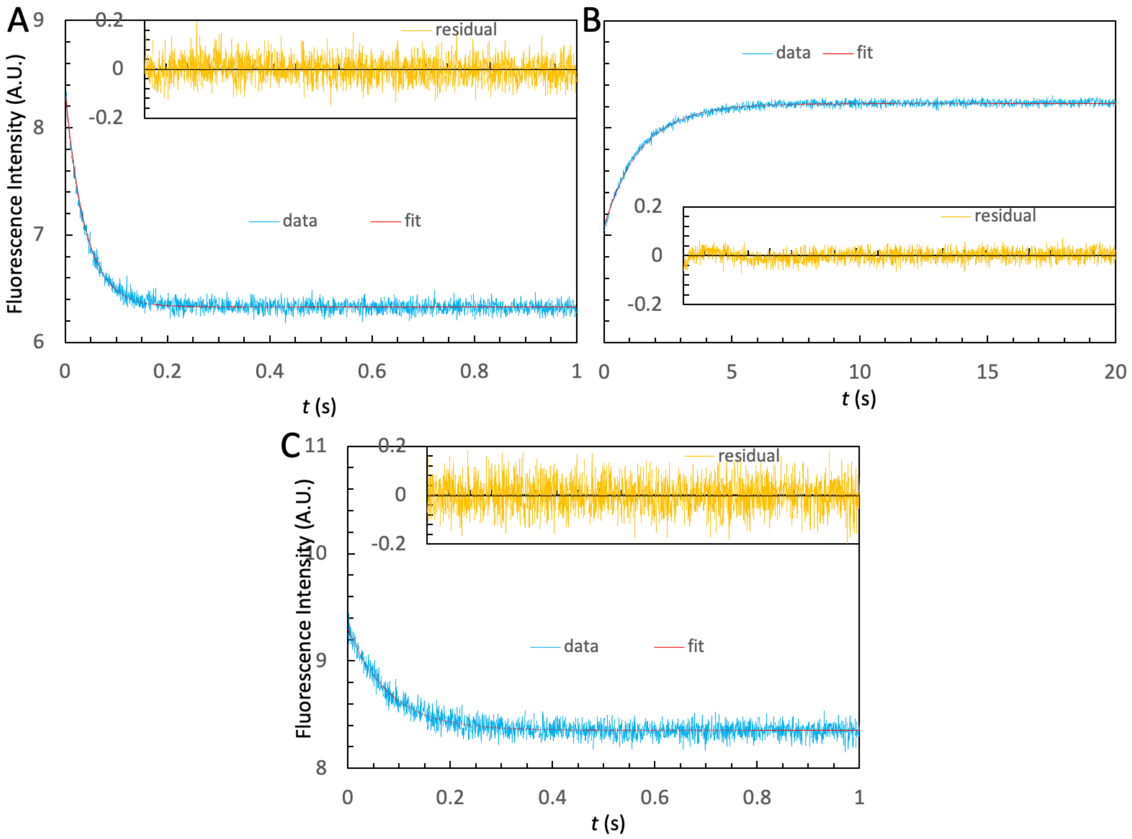

Fig. S1. Fitting of stopped-flow fluorescence intensity traces to a single exponential. (*A*) Data collected for the binding of wild-type Cdc42 and GBD at concentrations of 0.1 μM and 4.0 μM, respectively, in buffer. This fit yielded $k_{obs} = 25.5\ s^{-1}$. (*B*) Data collected when 0.1 μM mantGppNHp·Cdc42 was pre-mixed with 1 μM GBD, and then competed by 20 μM GppNHp·Cdc42. The fit yielded $k_d = 0.68\ s^{-1}$. (*C*) Same as in (*A*), but in 2 M urea. The fit yielded $k_{obs} = 12.4\ s^{-1}$. Inset: residuals of the fits.

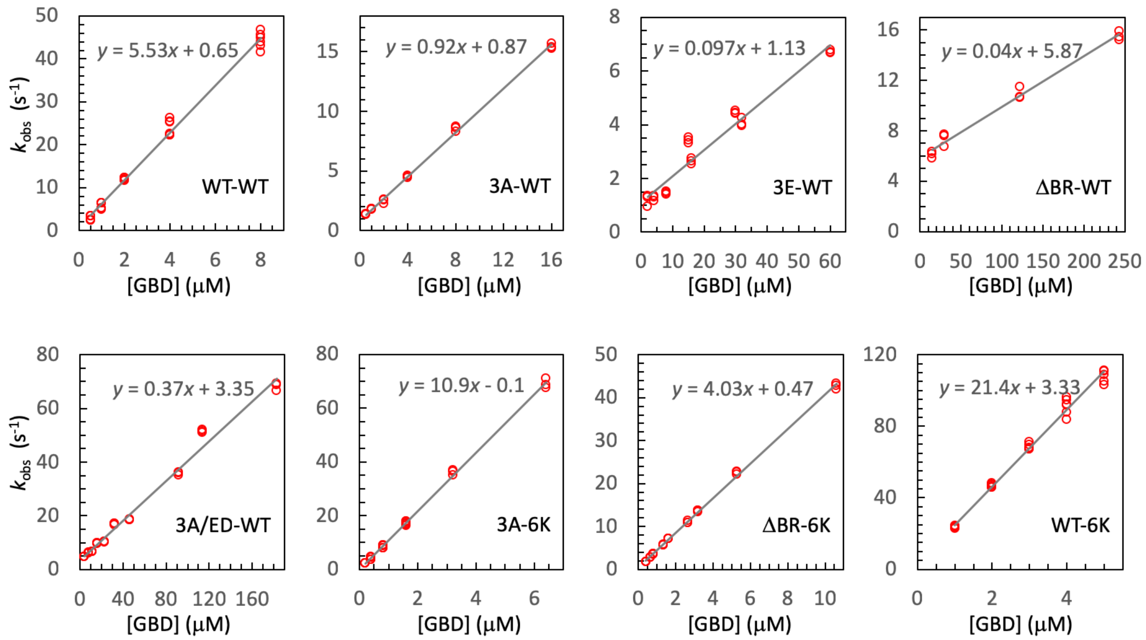

Fig. S2. Concentration dependences of k_{obs} for eight GBD-Cdc42 pairs in buffer. In the equation shown for the linear fit, x represents GBD concentration in μM, and y represents k_{obs} in s^{-1}.

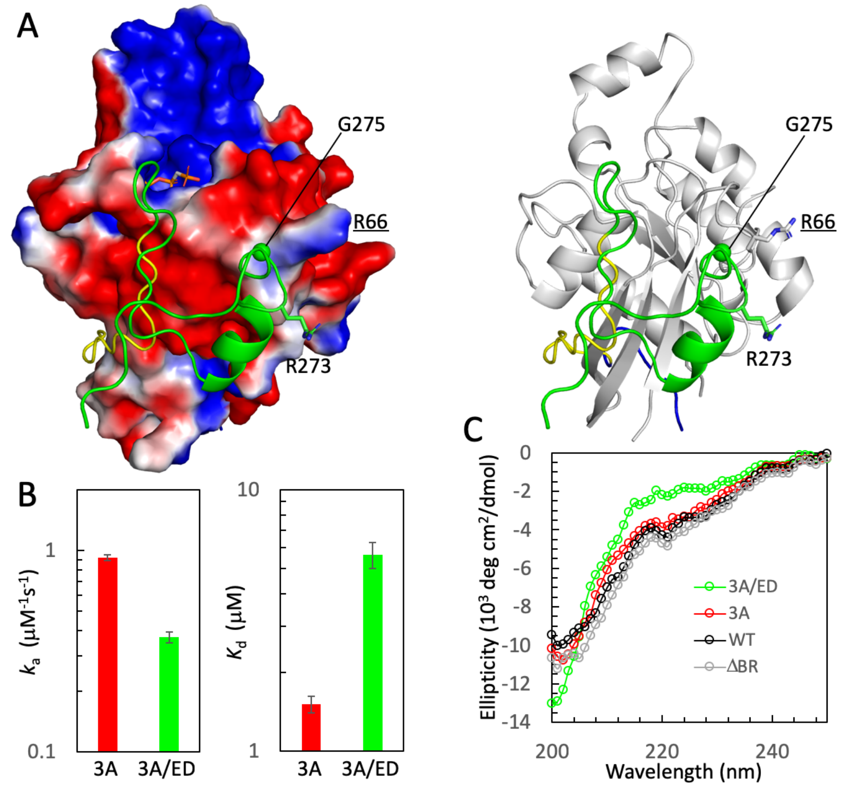

Fig. S3. GBD BR and Csub mutations and their effects on binding kinetic and thermodynamic properties. (*A*) Possible electrostatic attraction by Cdc42 Arg66 when two GBD Csub residues, Arg273 and Gly275, are mutated to Glu and Asp, respectively. Cdc42 is shown as either electrostatic surface (left panel) or cartoon in gray (right panel). (*B*) Association rate constants (k_a; left panel) and dissociation constants (K_d; right panel) for two GBD constructs, GBD_{3A} and $GBD_{3A/ED}$, interacting with $Cdc42_{WT}$. (*C*) Circular dichroism (CD) spectra of four GBD constructs, GBD_{WT}, GBD_{3A}, $GBD_{3A/ED}$, and $GBD_{\Delta BR}$. Mean ellipticity values per residue are shown as running averages at three neighboring wavelengths.

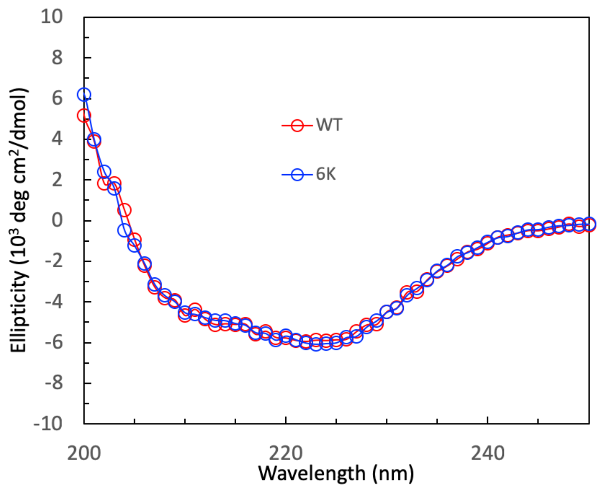

Fig. S4. Circular dichroism (CD) spectra of $Cdc42_{WT}$ and $Cdc42_{6K}$. Mean ellipticity values per residue at given wavelengths are shown.

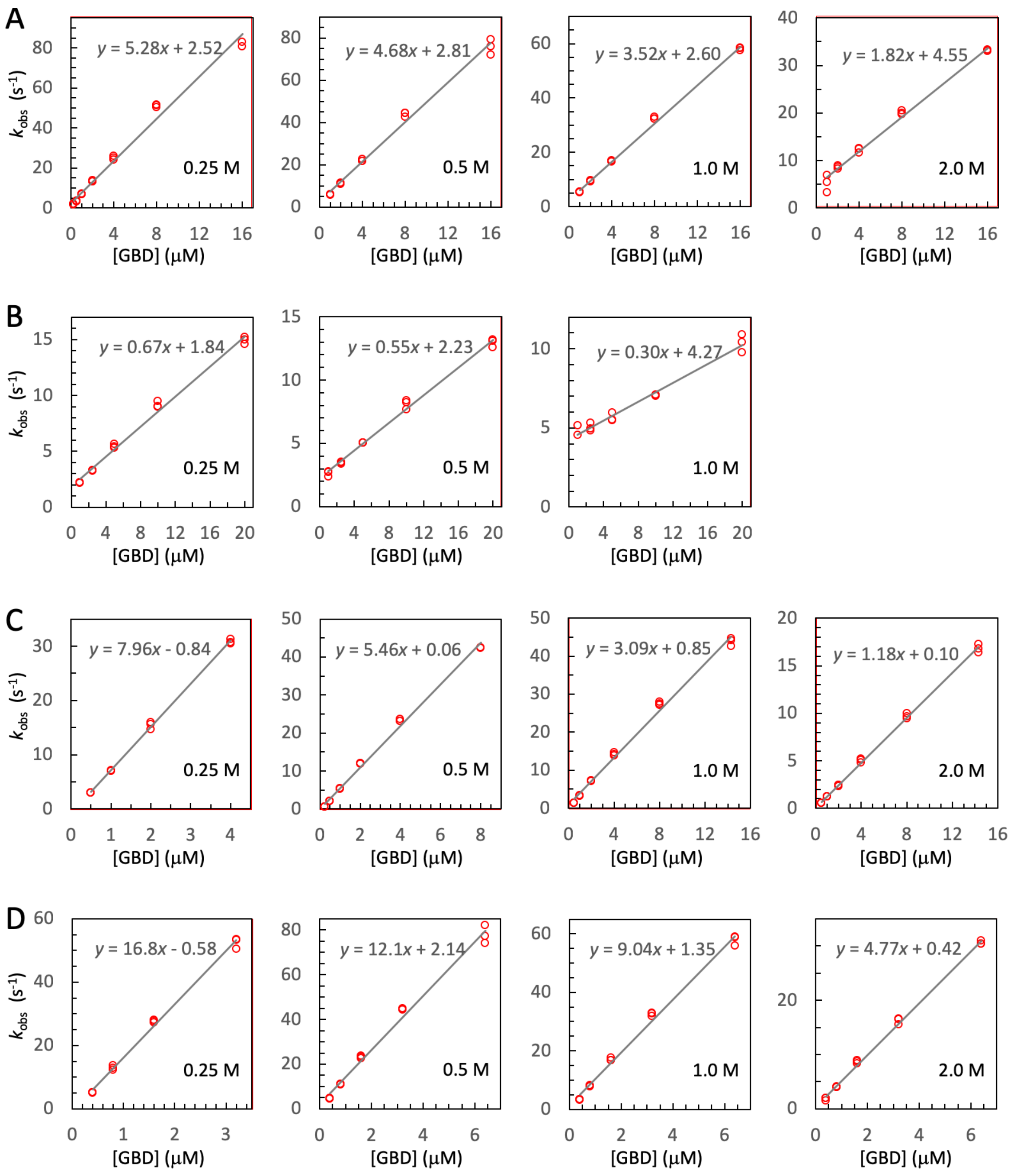

Fig. S5. Concentration dependences of k_{obs} for four GBD-Cdc42 pairs in urea at concentrations shown. (*A*) GBD_{WT} and $Cdc42_{WT}$. (*B*) GBD_{3A} and $Cdc42_{WT}$. (*C*) GBD_{3A} and $Cdc42_{6K}$. (*D*) GBD_{WT} and $Cdc42_{6K}$.

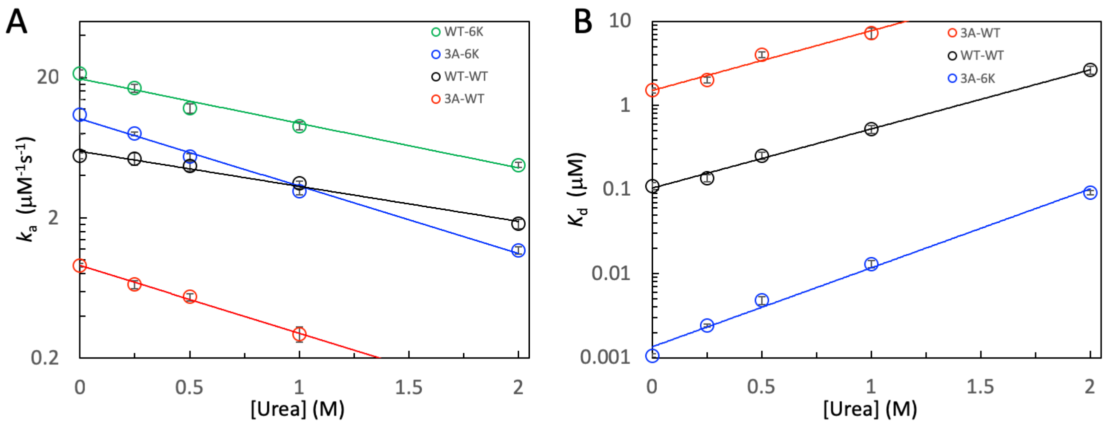

Fig. S6. Dependences of association rate constants and the dissociation constants of four GBD-Cdc42 pairs on urea concentration. Symbols are data and lines are linear fit of (*A*) ln k_a or (*B*) ln K_d. The slopes of the ln k_a fit are 0.58, 1.1, 1.1, and 0.73 M^{-1}, respectively, for GBD_{WT} and $Cdc42_{WT}$, GBD_{3A} and $Cdc42_{WT}$, GBD_{3A} and $Cdc42_{6K}$, and GBD_{WT} and $Cdc42_{6K}$. In comparison, the slopes are 1.6, 1.6, and 2.2 M^{-1}, respectively, for GBD_{WT} and $Cdc42_{WT}$, GBD_{3A} and $Cdc42_{WT}$, and GBD_{3A} and $Cdc42_{6K}$.

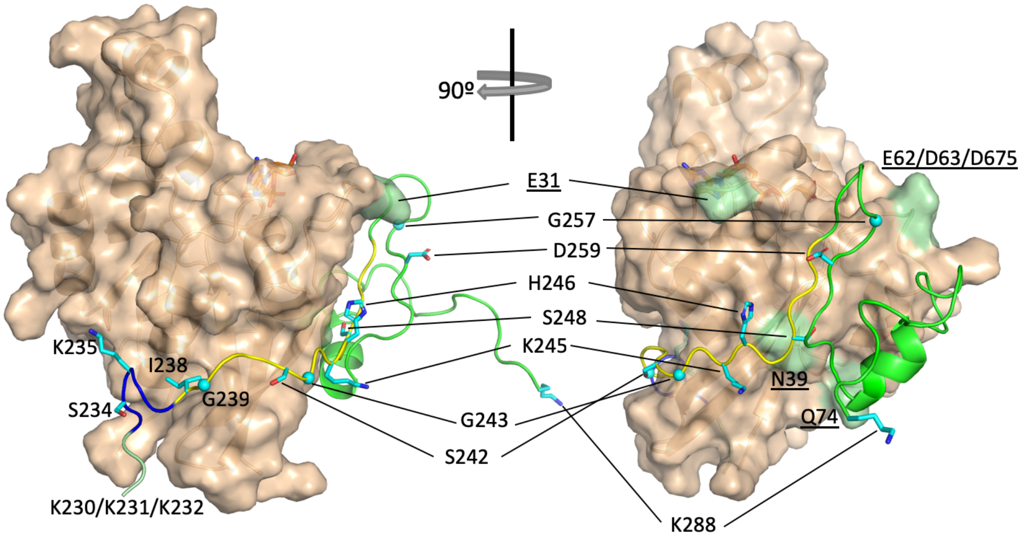

Fig. S7. Distribution of 12 GBD residues that had similar NMR peaks in the GBD_{WT}-$Cdc42_{WT}$ and GBD_{3A}-$Cdc42_{6K}$ complexes. The 12 residues are shown as cyan stick or sphere (for Gly). The 3A mutation sites in GBD and the 6K mutation sites in Cdc42 are also indicated, with pale green color.
